# Effect of three lactobacilli with strain-specific activities on the growth performance, faecal microbiota and ileum mucosa proteomics of piglets

**DOI:** 10.1186/s40104-017-0183-3

**Published:** 2017-06-09

**Authors:** Yating Su, Xingjie Chen, Ming Liu, Xiaohua Guo

**Affiliations:** 1 Provincial Key Laboratory for Protection and Application of Special Plants in Wuling Area of China, College of Life Science, South-Central University for Nationalities, No. 182, Minyuan Road, Hongshan District, Wuhan, Hubei Province 430074 China; 2 Guangxi Yang-Xiang Animal Husbandry Co. Ltd., Guigang, Guangxi Province 537100 China; 3 Beijing China-agri Hong-Ke Biotechnology Co., Ltd., Beijing, 102206 China

**Keywords:** Faecal microbiota, Growth, *Lactobacillus*, Mucosa proteomics, Probiotics

## Abstract

**Background:**

The beneficial effects of *Lactobacillus* probiotics in animal production are often strain-related. Different strains from the same species may exert different weight-gain effect on hosts in vivo. Most lactobacilli are selected based on their in vitro activities, and their metabolism and regulation on the intestine based on strain-related characters are largely unexplored. The objective of the present study was to study the in vivo effects of the three lactobacilli on growth performance and to compare the differential effects of the strains on the faecal microbiota and ileum mucosa proteomics of piglets.

**Methods:**

Three hundred and sixty piglets were assigned to one of four treatments, which included an antibiotics-treated control and three experimental groups supplemented with the three lactobacilli, *L. salivarius* G1-1, *L. reuteri* G8-5 and *L. reuteri* G22-2, respectively. Piglets were weighed and the feed intake was recorded to compare the growth performance. The faecal lactobacilli and coliform was quantified using quantitative PCR and the faecal microbiota was profiled by denaturing gradient gel electrophoresis (DGGE). The proteomic approach was applied to compare the differential expression of proteins in the ileum mucosa.

**Results:**

No statistical difference was found among the three *Lactobacillus*-treated groups in animal growth performance compared with the antibiotics-treated group (*P* > 0.05). Supplementation of lactobacilli in diets significantly increased the relative 16S rRNA gene copies of *Lactobacillus* genus on both d 14 and d 28 (*P* < 0.05)., and the bacterial community profiles based on DGGE from the lactobacilli-treated groups were distinctly different from the antibiotics-treated group (*P* < 0.05). The ileum mucosa of piglets responded to all *Lactobacillus* supplementation by producing more newly expressed proteins and the identified proteins were all associated with the functions beneficial for stabilization of cell structure. Besides, some other up-regulated and down-regulated proteins in different *Lactobacillus*-treated groups showed the expression of proteins were partly strain-related.

**Conclusions:**

All the three lactobacilli in this study show comparable effects to antibiotics on piglets growth performance. The three lactobacilli were found able to modify intestinal microbiota and mucosa proteomics. The regulation of protein expression in the intestinal mucosa are partly associated with the strains administrated in feed.

## Background

As living microorganisms, probiotics act in the intestine to modulate the host microbiota [1]. Among the strains of probiotics, lactic acid bacteria (LAB), especially from *Lactobacillus* and *Bifidobacteria* species, are recognized as one of the main sources and are widely used in food, drugs and feed additives as intestinal flora improvers [2]. In animal production, probiotics are expected to improve performance and to produce high-qualified meat without drug residues as an alternative to antibiotics [3].

Generally, *Lactobacillus* species selected for probiotics are highly diverse in the phenotypic and genetic characteristics [4]. Different strains may exert different weight-gain effect on hosts in vivo even if in the same species. Million et al. assessed the effect of lactobacilli-containing probiotics on weight based on 51 studies on farm animals and suggested that the weight-gain effect was greatly associated with strains of the genus [3]. Simon et al. showed similar results after summarizing above 20 published papers on lactobacilli used in feed additives [5]. The phenomena suggest that *Lactobacillus* strains may benefit their hosts through different mechanisms and more work should be done to explore the relationship between the choice of strains and their in vivo behaviours [6].

Nowadays the selection of *Lactobacillus* is often based on the strains’ activities in vitro, which is expected to show corresponding effectiveness in vivo. The strains with bacteriocin-producing activity showed specific anti-infective effect in the gut [7]. The strains with enzyme activities including amylase, protease and α-galactosidase had the potential to stimulate feed digestion [8–10]. However, the gut ecosystem was so complicated and the in vivo activities often depended on the strains’ survival and metabolism in the gastrointestinal tract (GIT) [10]. In our previous studies, three *Lactobacillus* strains (*Lactobacillus salivarius* G1-1, *Lactobacillus reuteri* G8-5 and *Lactobacillus reuteri* G22-2) were selected from swine faeces for probiotic use. They shared strain-related in vitro functional properties, including antimicrobial activity, amylolytic activity and bile-salt-hydrolase activity, respectively [11]. Meanwhile, from the in vivo studies in rats, the three *Lactobacillus* species showed some similar beneficial effects and some of the functionalities to rats were strain-specific [12]. When used in swine nutrition, the lactobacilli were hypothesized to interact with the intestinal flora and with the host mucosa, which might be associated with the mechanism of lactobacilli as probiotics. The objective of the present study was to study the in vivo effects of the three lactobacilli on growth performance and to compare the differential effects of the strains on the faecal microbiota and ileum mucosa proteomics of piglets.

## Methods

### *Lactobacillus* strains and freeze-dried powder preparation

Three strains, *L. salivarius* G1-1, *L. reuteri* G8-5 and *L. reuteri* G22-2 were isolated for probiotics based on the strain-specific functional properties in vitro [11]. All strains were incubated in DeMan Rogosa Sharp broth under anaerobic conditions at 37 °C for about 24 h. The microbial cells were collected by centrifugation at 11,000×g for 10 min, washed twice and mixed with protective additives for freeze-drying. The freeze-dried sample was smashed and diluted with dextrin as a carrier. The concentration of viable cells from each strain was determined by agar-plate assay and adjusted to 0.5 × 10^9^ colony forming unit per gram (CFU/g) by the carrier before animal trial.

### Animals, diets, experimental design and sampling

Three hundred and sixty castrated male, crossbred (Landrace × Large White) piglets, 35–40 days old, were randomly assigned to one of four treatments, which included an antibiotics control (Group A) and three experimental groups supplemented with *L. salivarius* G1-1 (Group B), *L. reuteri* G8-5 (Group C) and *L. reuteri* G22-2 (Group D), respectively. The piglets were housed with 15 piglets per pen and six pens of piglets received each treatment (*n =* 6). The pigs had free access to feed and water throughout the feeding trial with the environmental temperature 25–28 °C. The diet composition was listed in Table 1. The diet in the antibiotics control was supplemented with 200 mg/kg flavomycin. The three experimental diets consisted of the basal diet supplemented with 200 mg/kg *Lactobacillus* powder (10^9^ CFU/kg of feed) from each strain.

**Table 1:** Basal diet formula and nutrient levels

Ingredients	Percentage, %	Nutrient levels	
Extruded corn, soybean and sorghum with the proportion (3:1:1)	30.37	DE, Mcal/kg	3.30
High protein flour	17.40	Crude protein, %	18.2
Extruded soybean	5.00	Crude fat, %	5.05
Concentrated soybean meal	8.00	Crude ash, %	5.69
Limestone	1.00	Crude fiber, %	1.79
Calcium phosphate	0.69	Ca, %	0.75
Diamond V XP Yeast Culture	0.50	Total phosphorus, %	0.54
Mineral premix	1.50	Salt, %	0.63
Vitamins premix	1.00		
Lysine, 98%	0.55		
N-carbamoylglutamate	0.06		
Threonine	0.32		
Methionine	0.26		
Skim milk powder	7.50		
Whey powder	12.50		
Fatty powder	3.33		
Proprietary milk substitute	5.00		
Dextrose	5.00		
Antibiotics or lactobacilli powder	0.02		
Total	100.00		

^a^ Vitamins provided per kilogram diets: vitamin A, 8000 IU; vitamin D_3_, 1800 IU; vitamin E, 30 IU; vitamin K_3_, 3.56 mg; vitamin B_1_, 1.8; vitamin B_2_, 6 mg; vitamin B_6_, 1.26 mg; vitamin B1_2_, 0.02 mg; folic acid, 0.3 mg; biotin, 0.44 mg; niacin, 32 mg; pantothenic acid, 15 mg

^b^ Minerals provided per kilogram diets: Cu, 250 mg; Fe, 130 mg; Zn, 130 mg; Mn, 60 mg; Se, 0.3 mg; I, 0.4 mg

Piglets were weighed and the feed intake was recorded during the trial term to calculate the average daily weight gain (ADG), average daily feed intake (ADFI) and feed conversion ratio (F:G). Fresh faecal samples (4–5 g) from 3–4 individual piglets were collected and pooled from three randomly chosen pens were collected for each treatment on d 14 and 28. The samples after collection were immediately stored at −20 °C until the molecular analysis for microbiota. At the end of the trial, three randomly chosen piglets were selected from each treatment and slaughtered for ileum sampling. About 20 cm ileum at the same place of each pig were rapidly cut and the chyme was washed out using sterile water. The mucosa was carefully scrapped by coverslips and kept in 1.5 mL Eppendorf tube. The samples were frozen immediately by liquid nitrogen and stored at −80 °C for proteomics analyses. All surgical and animal care procedures in the study followed the protocols approved by Experimental Animal Care and Use Guidelines (Chinese Science and Technology Committee, 1988).

### DNA extraction, real-time quantitative PCR and PCR-DGGE analyses

The total genomic DNA was extracted from faeces (about 1.0 g) based on the method of bead-beating and following phenol-chloroform extraction [13, 14]. Total lactobacilli and coliform were detected by real-time quantitative PCR, respectively. The lactobacilli were quantified using primer Lac1 (5′-AGCAGTAGGGAATCTTCCA-3′), and Lab0677 (5′- CACCGCTACACATGGAG −3′) [15]. Two primers, EcoliFimH2F (5′-AGCAGTAGGGAATCTTCCA-3′) and EcoliFimH2R (5′- TCATCCCTGTTATAGTTGYYGGTCT-3′) were used to amplify 16S rRNA gene of coliform [16]. The reverse transcription PCR (RT-PCR) system was quantified using the ABI 7500 system (Applied Biosystems, US). The optimum thermal cycles were performed as follows: pre-denaturation at 95 °C for 10 min, 40 cycles of 95 °C for 15 s and 60 °C for 1 min, and followed by the stage of melting curve. The relative 16S rRNA gene copies were calculated through the 2^−ΔΔCT^ method according to the report of Livak [17]. The results were compared based on the three paralleled values of faeces from each treatment.

A set of universal primers, U968-GC (5′-CGCCCGGGGCGCGCCCCGGGCGGGGCGGGGGCACGGGGGGAACGCGAAGAACCTTAC-3′), L1401 (5′-CGGTGTGTACAAGACCC-3′) [18], Bact 1369 F (5′-CGGTGAATACGTTCYCGG-3′), and 1492R (5′-GGWTACCTTGTTACGACTT-3′) [19] were employed to amplify the total bacteria. The amplicons were separated by DGGE according to the specification as described previously [20]. Briefly, DGGE was performed in 8% polyacrylamide gels (acrylamide-bis, 37.5:1). The gels with a 38–51% denaturing gradient was used for the separation of PCR products based on the primers U968-GC and L1401, while gradients of 30–45% were applied for the separation of the Bact 1369 F and 1492R generated amplicons. The electrophoresis procedures were performed at 70 V for 16 h at 60 °C and the gel was finally stained with SYBR Green I for 30 min after electrophoresis. The DGGE gels were scanned using an image scanner and analysed with Bio-rad gel imaging system through Quantity One software (Version 4.6.2).

The similarities among DGGE profiles were determined by Dice coefficient based on the unweighted pair group method with arithmetic average (UPGMA) clustering algorithm [21]. The faecal samples from the antibiotics group were evenly mixed and conducted for DGGE profiles used as the control band. The bands from three paralled faecal samples of each *Lactobacillus* group were profiled and compared with the control band (*n =* 3).

### 2-dimensional electrophoresis (2-DE), image analysis and protein identification

Isoelectric focusing (IEF) was performed using immobilized pH gradient (IPG) Strips (pH 4–7; 7 cm long; Pharmacia Biotech.). Samples were diluted with IEF buffer containing 7 mol/L urea, 2 mol/L thiourea, 4% CHAPS, 20 mmol/L Tris–HCl, pH 8.5, 20 mmol/L DTT, 0.5% carrier ampholyte (pH 4–7) and a trace of bromophenol blue. The desired protein amount in buffer was 50 μg. After equilibration, the immobilized pH gradient strips were loaded onto 12.5% (w/v) homogeneous acrylamide gels and sealed with 1% (w/v) agarose. The electrophoretic separation of proteins was conducted as described previously [22, 23]. Upon completion of 2-dimensional SDS-PAGE, the gels were stained by silver or Coomassie Brilliant Blue G-250. The high-resolution gel images (200 dpi) from silver-stained gels were obtained using an image scanner (Powerlook1100, UMAX) for image analysis. The gels stained by silver were run in triplicate, and spots that appeared consistently in all three runs were selected for analysis. Spot detection and analysis were performed using the PDQuest version 6.1 software (Bio-Rad) according to the protocols provided by the manufacturer. Some differentially expressed protein spots with 3.0-fold differences in volume detected by the software were selected for protein identification. The protein spots of interest were confirmed in the Coomassie Brilliant Blue stained gels and manually excised for the treatment of digestion by trypsin. The matrix-assisted laser desorption/ionization time of flight mass spectrometry (MALDI-TOF MS) was used for protein identification as described by early reports [24, 25]. The peptide fragments produced from each protein spot were employed to produce peptide-mass mapping (PMM) data. The protein identification was carried out by peptide mass fingerprinting (PMF) analysis through the MASCOT server (www.matrixscience.com; Matrix Science, UK). The search parameters were as follows, database: Swiss-Prot Sus (34361 sequences); species: sus; enzyme: trypsin; fixed modifications: carbamidomethylation; variable modifications: oxidation (M). The gene name, accession code and function of each protein were determined using the Mascot V2.1 software protein database search engine and the Swiss-Prot Sus protein database.

### Statistical analyses

All quantitative data were expressed as the mean and standard deviation of replicates. The differences among antibiotics-treated and lactobacilli-treated groups were considered statistically significant at *P* < 0.05 using one-way analysis of variance (One-way ANOVA) through JMP software (JMP; SAS Institute Inc., Cary, NC). 0.5 < *P* < 0.1 was considered a trend towards significance.

## Results

### Growth performance

Over the 4-week feeding trial, there was no statistical difference in ADG, ADFI and F:G between piglets supplemented with lactobacilli and the antibiotics group (Table 2). Among the three *Lactobacillus* groups, the diet containing *L. reuteri* G8-5 tended to show lower ADG and ADFI than that of the other two *Lactobacillus* groups (0.5 < *P* < 0.1).

**Table 2:** The effect of three lactobacilli on the growth performance of weaned piglets during a 4-week feeding trial

Treatments	Antibiotics (A)	*L. salivarius* G1-1 (B)	*L. reuteri* G8-5 (C)	*L. reuteri* G22-2 (D)	*P*-value					
					A vs. B	A vs. C	A vs. D	B vs. C	B vs. D	C vs. D
Initial body weight, kg	7.44 ± 1.22	7.51 ± 1.00	7.56 ± 0.92	7.51 ± 0.92	0.913	0.871	0.922	0.957	0.992	0.949
Final body weight, kg	14.48 ± 1.19	14.81 ± 0.56	13.88 ± 1.13	14.71 ± 1.87	0.727	0.516	0.807	0.325	0.916	0.376
ADG, g/d	270.9 ± 17.1	280.3 ± 20.5	243.3 ± 15.8	276.9 ± 38.8	0.603	0.141	0.743	0.056	0.846	0.080
ADFI, g/d	408.6 ± 33.4	422.8 ± 15.4	338.5 ± 36.9	418.1 ± 20.5	0.486	0.331	0.639	0.109	0.817	0.161
F:G	1.51 ± 0.13	1.51 ± 0.08	1.60 ± 0.09	1.54 ± 0.11	0.976	0.338	0.859	0.337	0.872	0.420

Values are means ± S.D, *n =* 6

### Relative 16S rRNA gene copies by RT-PCR

**Fig. 1:**
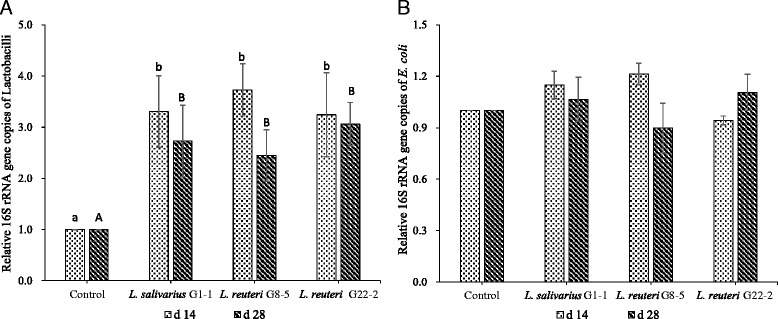
Effect of three *Lactobacillus* strains on the fecal relative 16S rRNA gene copies of lactobacilli (**a**) and *E. coli* (**b**), respectively on d 14 and d 28. ^ab^ mean in the same column from the result on d 14 with different scripts differ significantly (*P* < 0.05); ^AB^ means in the same column from the result on d 28 with different scripts differ significantly (*P* < 0.05)

A comparison of the relative 16S rRNA gene copies of *Lactobacillus* and coliform in faeces on d 14 and d 28 was shown in Fig. 1. Supplementation of lactobacilli in diets significantly increased the counts of *Lactobacillus* genus on both d 14 and d 28 compared with the antibiotics group (*P* < 0.05). However, no significant difference in the relative 16S rRNA gene copies of coliform was observed in all groups (*P* > 0.05).

### PCR-DGGE profiles

**Fig. 2:**
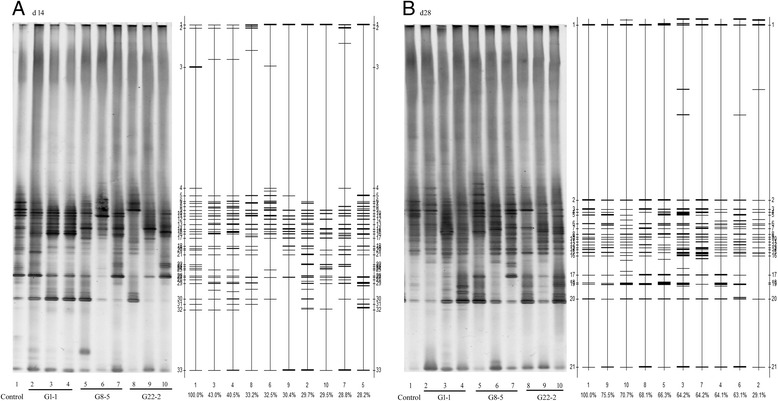
PCR-DGGE DNA profiles of the 16S rRNA of microbiota in faces of weaned pigs at d 14 (**a**) and d 28 (**b**) during a 4-week feeding trial

The representative DGGE profiles were presented in Fig. 2. The DGGE patterns were transformed into graphs by the Bio-Rad Quantity One^TM^ software, which calculated the Dice similarity among lanes (Fig. 2). The similarities among four treatments on d 14 and d 28 were listed in Table 3. On d 14, the dendrogram based on the banding patterns showed low similarities and the bacterial community profiles form the lactobacilli were distinctly different from the antibiotics group (*P* < 0.05). Meanwhile, the similarities in *L. reuteri* G8-5 group were significantly lower than those in *L. salivarius* G1-1 group (*P* < 0.05). On d 28, the percentage of similarity in all *Lactobacillus* groups increased but was still significantly lower than that of antibiotics group. There was no marked difference in similarities in all *Lactobacillus*-treated groups on d 28 (*P* > 0.05).

**Table 3:** Effect of three *Lactobacillus* strains on the similarities among digitalized DGGE profiles of PCR-amplified 16S rRNA from fecal DNA after Bio-Rad Quantity One software comparison

	Similarity, %	
Treatments	d 14	d 28
Antibiotics	100.00 ± 0.00^a^	100.00 ± 0.00^a^
*L. salivarius* G1-1	37.73 ± 7.07^b^	52.47 ± 20.24^b^
*L. reuteri* G8-5	29.83 ± 2.33^c^	64.53 ± 1.63^b^
*L. reuteri* G22-2	31.03 ± 1.93^bc^	71.43 ± 3.75^b^

Values are means ± S.D, *n =* 3. ^a, b, c^ Mean in a same column with different superscripts differ significantly (*P* < 0.05)

### 2-DE profiles of differentially expressed proteins

**Fig. 3:**
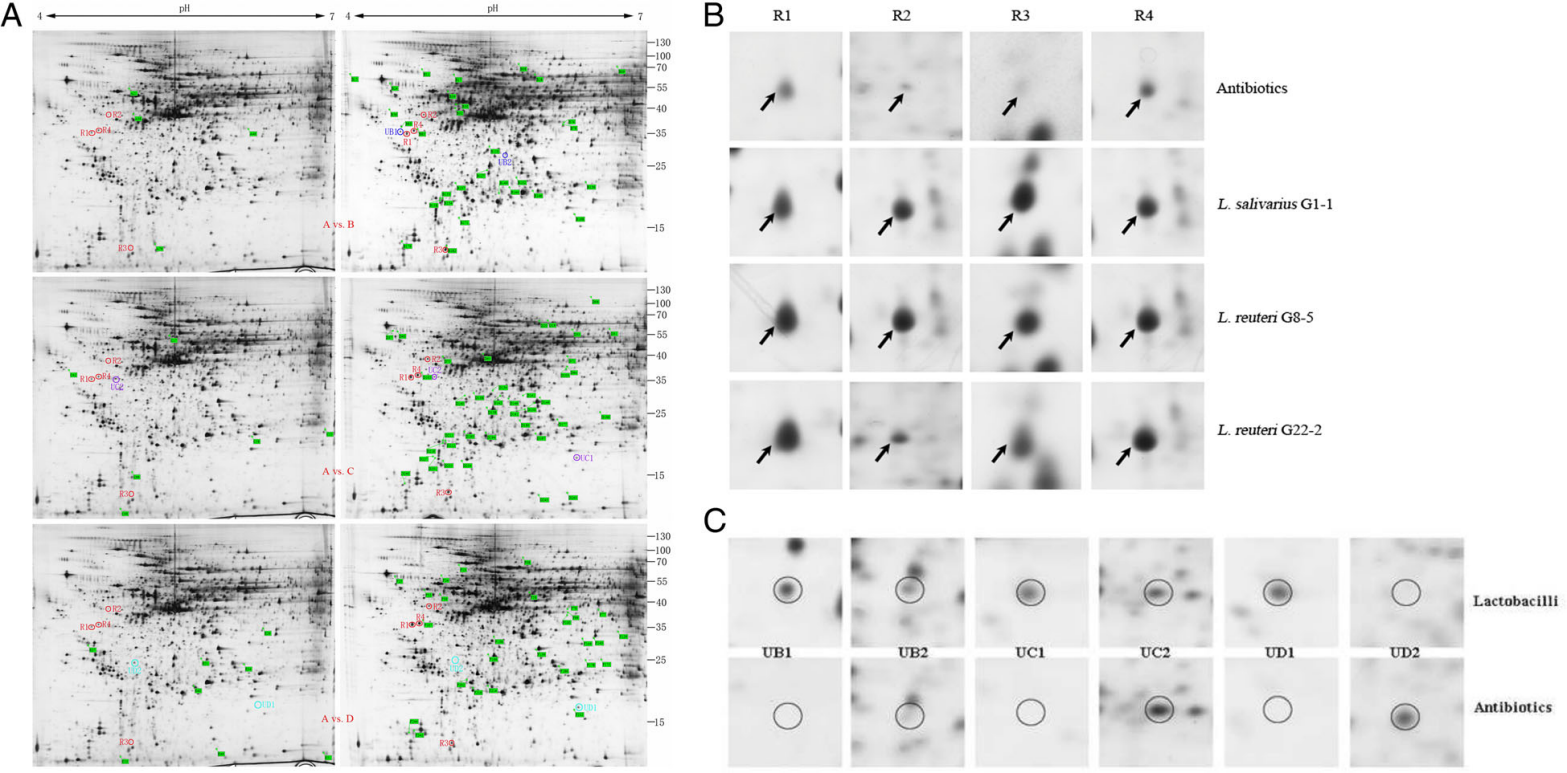
Representative 2-DE profiles of differentially expressed proteins in the small intestinal mucosa of piglets administrated by lactobacilli or antibiotics. (**a**): Distribution of differentially expressed proteins in antibiotics group (**a**) and each *Lactobacillus* group (**b**, **c**, **d**; G1-1, G8-5, G22-2, respectively); (**b**): Up-regulated protein spots in all *Lactobacillus*-treated piglets compared with antibiotics-treated piglets; (**c**): Differentially expressed proteins spots varying from *Lactobacillus* and antibiotics-treated piglets

By comparing the 2-DE profiles of differentially expressed proteins in the ileum of piglets between the antibiotics-treated and *Lactobacillus*-treated groups, supplementation of lactobacilli significantly increased the counts of newly expressed proteins. 4, 6 and 8 new proteins were expressed only in the antibiotics group compared with the three *Lactobacillus* groups, respectively. Nevertheless, 32, 40 and 27 new proteins only existed in the three *Lactobacillus* groups compared with the antibiotics group, respectively (Fig. 3a). Among the differentially expressed proteins, 4 protein spots which were up-regulated in all the *Lactobacillus*-treated groups were selected for the identification by MALDI-TOF. These proteins included tropomyosin beta chain (TPM2, Spot R1), vimentin (VIM, Spot R2), keratin type I cytoskeletal 19 (KRT19, Spot R3), tropomyosin alpha-1 chain (TPM1, Spot R4) (Table 4; Fig. 3b). Other six protein spots were chosen because they were specifically affected by different *Lactobacillus* strains (Table 4; Fig. 3c). The proteins in *L. salivarius* G1-1 group included the up-regulation of phosphatidylinositol 4,5-bisphosphate 3-kinase catalytic subunit gamma isoform (PIK3CG, Spot UB1) and cofilin-1 (CFL1, Spot UB2), which were only detectable in *L. salivarius* G1-1-treated group. The proteins expressed in *L. reuteri* G8-5 group included the up-regulation of Rho GDP-dissociation inhibitor 2 (ARHGDIB, Spot UC1; only detectable in lactobacilli group) and the down-regulation of nucleophosmin (NPM1, Spot UC2). The proteins in *L. reuteri* G22-2 group included the up-regulation of Rho GDP-dissociation inhibitor 2 (ARHGDIB, Spot UD1; only detectable in lactobacilli-treated group) and the down-regulation of actin cytoplasmic 1 (ACTB, UD2; only detectable in antibiotics group).

**Table 4:** Differentially expressed proteins in the ileum mucosa of piglets supplemented by three lactobacilli in diets compared with antibiotics

Category	Spot No.^a^	Gene	Accession code	Protein	Expression change (Lactobacilli VS. Antibiotics)	Score^b^	Putative function
Lactobacilli-insensitive spots compared with antibiotics	R1	*TPM2*	F1SG00	Tropomyosin beta chain	Up-regulation	261	Stabilizing cytoskeleton actin filaments
	R2	*VIM*	P02543	Vimentin	Up-regulation	138	Maintaining cell shape, integrity of the cytoplasm, and stabilizing cytoskeletal interactions
	R3	*KRT19*	F1S0J8	Keratin type I cytoskeletal 19	Up-regulation	185	Maintaining structural integrity of epithelial cells
	R4	*TPM1*	F2Z5B6	Tropomyosin alpha-1 chain	Up-regulation	277	Stabilizing cytoskeleton actin filaments
Lactobacilli-related spots compared with antibiotics	UB1(G1-1)	*PIK3CG*	O02697	Phosphatidylinositol 4,5-bisphosphate 3-kinase catalytic subunit	Only detectable in lactobacilli	137	Maintaining structural and functional integrity of epithelia
	UB2(G1-1)	*CFL1*	P10668	Cofilin-1	Only detectable in lactobacilli	121	Regulation of cell morphology and cytoskeletal organization
	UC1(G8-5)	*ARHGDIB*	F1SQW8	Rho GDP-dissociation inhibitor 2	Only detectable in lactobacilli	109	Small GTPase regulator activity receptor binding;
	UC2(G8-5)	*NPM1*	F1RRY2	Nucleophosmin	Down-regulation	99	Ribosome biogenesis and transport
	UD1(G22-2)	*ARHGDIB*	F1SQW8	Rho GDP-dissociation inhibitor 2	Only detectable in lactobacilli	116	Small GTPase regulator activity receptor binding;
	UD2(G22-2)	*ACTB*	Q6QAQ1	Actin cytoplasmic 1	Only detectable in antibiotics	139	Involved in cell motility, structure, and integrity

^a^ Spot No. refers to protein spot numbers that were labeled in Fig. 3

^b^ Protein score generated by MS identification platform MASCOT; a score > 65 is considered significant

## Discussion

The supplementation of lactobacilli in animal diets affects gastrointestinal tract health and growth performance of piglets [1, 5]. However, different *Lactobacillus* strains used as probiotics may achieve the beneficial effects on hosts through different mechanisms [12, 26]. The present study was conducted to compare the different efficacies among three lactobacilli with strain-specific activities in growth performance, faecal microbiota and ileum mucosa proteomics of piglets.

No significant differences in growth performance among *Lactobacillus*-treated groups were observed compared with the antibiotics-treated group. The result showed that all the three lactobacilli had the same potential as alternative to antibiotics in feed. However, among the three lactobacilli, the supplementation of *L. reuteri* G8-5 was the least effective in enhancing the growth performance of piglets, which was in line with the previous study in the rat experiment [12]. The reason is probably associated with the strain’s lower antimicrobial activity compared with the other two strains, which was reported in the previous study [11].

Increased lactobacilli in faeces from lactobacilli-treated piglets on both d 14 and d 28 in this study verified the ability of the three lactobacilli to maintain the balance of microbiota, which was one of the possible mechanisms of lactobacilli as probiotics in vivo [27]. Meanwhile, the modulation of intestinal microbiota by lactobacilli might be strain-insensitive since all the three lactobacilli used in the study showed the same ability as intestinal flora improvers. No difference in coliform counts was observed in whole feeding period compared with the antibiotics group. The result suggested the antibiotics used in the study and lactobacilli had the similar resistance to pathogens and kept them in low level in the gastrointestinal tract. It is assumed that the increasing intestinal microbial abundance caused by antibiotics or lactobacilli has more power to resist the disruption of microbial balance [28, 29]. Further analysis on the microbiota in the gastrointestinal tract treated by lactobacilli and antibiotics by PCR-DGGE was investigated for the comparison of microbial diversity. On d 14 and d 28, the similarities in all *Lactobacillus*-treated groups were significantly different from the antibiotics-treated group. The results suggested that the mechanisms of antibiotics and lactobacilli on regulating intestinal microbiota were through different ways and lactobacilli contributed to comparatively complex bacterial community. Some similar results were also shown in other reports [30, 31]. The results in Table 4 and Fig. 2 showed the discrepancy in similarities between lactobacilli and antibiotics treatments tended to decrease from d 14 to d 28. This indicates the bacterial diversity tended to be stable and not sensitive to extraneous drugs or introduced bacteria during animals’ growth. The significantly lower Dice similarity in *L. reuteri* G8-5 compared with *L. salivarius* G1-1 was observed in this study, and the result was in line with that in the growth performance.

Proteomics play an important role in the assessment of specific health-promoting activities exerted by *Lactobacillus* species [32, 33]. The ileum mucosa samples were collected to compare the differentially expressed proteins through 2-DE profiles. From the result in Table 4, the supplementation of lactobacilli all greatly increased the number of expressed protein spots compared with the antibiotics group. Similar result was also observed in the study of Wang et al. [32]. Up-regulation of four proteins including TPM2, VIM, KRT19 and TPM1 in all three *Lactobacillus* groups are all associated with the functions of maintaining and stabilizing cell structure and stabilization. The four proteins were inferred to be *Lactobacillus*-insensitive, and the mutual mechanisms for *Lactobacillus* as probiotics were to enhance the expression of proteins beneficial for stabilization of cell structure. Both TPM1 and TPM2 bind to actin filaments and up-regulation of the two proteins benefit to stabilizing cytoskeleton actin filaments [34]. Meanwhile, increased level of VIM is responsible for maintaining cell shape, integrity of the cytoplasm, and stabilizing cytoskeletal interactions [35]. The up-regulation of KRT19 is responsible for the structural integrity of epithelial cells [36]. The increased expression of KRT19 in lactobacilli groups can contribute to more opportunities for living cells to adhere to the epithelial and exclusively inhibit pathogen infection [37, 38]. Similar result was also observed in the study of Wang et al. [32], in which KRT10 was higher in the intestinal mucosa of piglets supplemented with *L. fermentum* I5007 compared with that in antibiotics piglets [32]. Both KRT10 and KRT19 belong to the keratin family which are intermediate filament proteins responsible for the structural integrity of epithelial cells [36].

There were six extra proteins differently expressed in different *Lactobacillus* groups, which were inferred to be *Lactobacillus*-related. The different expression of protein might be caused by the characters of specific strains. In the groups of *L. salivarius* G1-1, two proteins, PIK3CG and CFL1, detected only in *Lactobacillus* group were also associated with cell structure and stability.

ARHGDIB was only detectable in the ileum mucosa of piglets in response to the supplementation of both *L. reuteri* G8-5 and *L. reuteri* G22-2. The high expression of the protein enhances the recycling and distribution of activated Rho GTPases in the cell and play a role in regulating cell motility through the modulation of Rho proteins [39]. NPM1 help cells survive environmental stresses, such as drug attack [40]. Up-regulation of NPM1 in antibiotics might be associated with the intake of flavomycin. The increase in ACTB found in vivo would indicate drastic oxidative modification leading to functional impairments [41], which might be the side-effect of antibiotics supplemented in feed. More experiments are needed in order to document the potential beneficial effects of the lactobacilli strains for the piglets, notably in terms of mucosal health.

## Conclusions

In conclusion, this study provides a comprehensive comparison of three lactobacilli with strain-specific activities through the supplementation in piglet diets. All the three lactobacilli show the potential as alternatives to antibiotics and no statistical difference in animal growth performance compared with the antibiotics group. Supplementation of lactobacilli in diets could significantly increase the relative 16S rRNA gene copies of lactobacilli genus on both d 14 and d 28, and the bacterial community profile based on PCR-DGGE from the lactobacilli are distinctly different from the antibiotics group. The ileum mucosa piglets respond to all lactobacilli supplementation by more newly expressed proteins and the identified proteins are all associated with the functions beneficial for stabilization of cell structure. Besides, some other up-regulated and down-regulated proteins in different *Lactobacillus* groups showed the expression of proteins were partly strain-related.

This comparative study helps to explore the mutual mechanisms for *Lactobacillus* as probiotics on altering intestinal abundance of microbiota and expression of mucosa proteins in piglets and provides information for strain-specific screening in application.

## Abbreviations

2-DE: 2-dimensional electrophoresis
ACTB: Actin cytoplasmic 1
ADFI: Average daily feed intake
ADG: Average daily weight gain
ARHGDIB: Rho GDP-dissociation inhibitor 2
CFU: Colony forming unit
DGGE: Denaturing gradient gel electrophoresis
DNA: Deoxyribose nucleic acid
F:G: Feed conversion Ratio
GIT: Gastrointestinal tract
IEF: Isoelectric focusing
IPG: Immobilized pH gradient
KRT19: Keratin type I cytoskeletal 19
LAB: Lactic acid bacteria
MALDI-TOF MS: Matrix-assisted laser desorption/ionization time of flight mass spectrometry
NPM1: Nucleophosmin
PCR: Polymerase chain reaction
PIK3CG: Phosphatidylinositol 4,5-bisphosphate 3-kinase catalytic subunit gamma isoform
PMF: Peptide mass fingerprinting
PMM: Peptide-mass mapping
rRNA: Ribosomal ribonucleic acid
RT-PCR: Reverse transcription-polymerase chain reaction
SDS-PAGE: Sodium dodecyl sulfate-polyacrylamide gel electrophoresis
TPM1: Tropomyosin alpha-1 chain
TPM2: Tropomyosin beta chain
UPGMA: Unweighted pair group method with arithmetic average
VIM: Vimentin
